# Use of a handheld communication tool for children and youths during dental procedures: a two-site controlled intervention study

**DOI:** 10.2340/aos.v85.45760

**Published:** 2026-03-31

**Authors:** Elisabeth Ørskov Rotevatn, Emilie Stensaker Paz, Louise Sandal Løkeland, Frode Guribye, Grete Olin Engan, Cecilie Gudveig Gjerde, Torgils Lægreid, David N. Breidablik Vatne, Unnur Bergmann, Lars Jørgen Rygh, Mette Engan

**Affiliations:** a Department of Clinical Science, Faculty of Medicine, University of Bergen, Bergen, Norway; b Department of Anesthesia and Intensive Care, Haukeland University Hospital, Bergen, Norway; c Children and Youth Clinic, Haukeland University Hospital, Bergen, Norway; d Department of Information Science and Media Studies, University of Bergen, Bergen, Norway; e Sogndal Dental Clinic, Vestland County Municipality, Sogndal, Norway; f Department of Oral and Maxillofacial Surgery, Institute of Clinical Dentistry, University of Bergen, Bergen, Norway; g Center for Translational Oral Research, Department of Clinical Dentistry, Medical Faculty, University of Bergen, Bergen, Norway

**Keywords:** pediatric dental care, procedural coping, dental fear and anxiety, nonverbal communication, digital health

## Abstract

**Background:**

Effective communication between dental professionals and patients is essential for quality care. We aimed to evaluate whether *Grasp*, a novel handheld squeezable device providing real-time visual and auditory feedback to signal discomfort, improved communication between patients and their dentists during dental procedures.

**Materials and methods:**

Patients aged 6–25 years were recruited from two dental clinics in Norway. Participants were divided into two groups: One received standard care, the other used *Grasp* in addition to standard care. All patients – and additionally caregivers of those under 16 – completed pre- and post-treatment questionnaires, rating communication on a 1–10 scale. Multiple linear regression analyses were conducted to evaluate the intervention’s effect, adjusting for baseline scores.

**Results:**

A total of 121 patients (58% females; median age 16 years) participated, with 60 using *Grasp*. Patients using *Grasp* reported greater confidence that the dentist understood their feelings, recognized when they wanted to stop, and found it easier to speak up during treatment (β = 2.10–2.54, *p* < 0.05). The effect of *Grasp* was more pronounced for participants reporting lower baseline confidence in communication with their dentist.

**Conclusions:**

*Grasp* appeared to improve communication regarding discomfort during dental procedures, particularly for those who initially reported lower levels of communication confidence.

## Introduction

The World Dental Federation emphasizes effective communication between oral healthcare providers and patients as a fundamental component of high-quality care [1]. Individuals seeking dental care value clinicians who demonstrate attentiveness, empathy, respect, and actively engage patients in decision-making processes [2]. Clear and structured communication can improve health outcomes, enhance patient satisfaction, and promote adherence to treatment plans [2].

Perceived control is a key resource when dealing with stressful situations [3]. Patients’ perceived lack of control during dental procedures has been found to be associated with avoidance behaviors and heightened distress [4–6]. Dental fear and anxiety, which often emerge during childhood or adolescence [7–10], are strongly linked to this sense of lost control [4–6, 10]. Strengthening patients’ sense of control through effective communication – by enabling patients to understand, anticipate, and influence what happens during treatment – is therefore considered an important strategy for improving the dental experience in both adult and pediatric dentistry [5, 11, 12]. However, communication during dental procedures remains inherently constrained by physical and environmental factors. Non-verbal communication strategies, such as agreed stop signals or eye contact, are sometimes used, and some digital tools have been developed to improve interaction [2, 13–16]. Yet, a peri-procedural communication tool for conveying discomfort with a suitable design for different age groups could further strengthen the patients’ sense of control and improve both satisfaction and overall quality of care.

*Grasp* is a digital handheld and squeezable device that provides real-time visual and auditory feedback to signal discomfort, designed to facilitate non-verbal communication between patients and dentists. The aim of this study was to examine whether the use of *Grasp* affected communication about peri-procedural discomfort between children and youths and their respective dentists across two clinical settings. We hypothesized that participants using Grasp would report higher post-treatment communication scores compared to those receiving standard care.

## Materials and methods

To explore the effect of using *Grasp* as a communication tool for children and youths during dental treatment, we designed a clinical performance study comparing a group of patients receiving standard care to an intervention group.

### Study population

Participants were recruited from two dental clinics: the Department of Clinical Dentistry, University of Bergen, Norway, and a public dental clinic in Sogndal, Vestland County Municipality, Norway.

Inclusion criteria were slightly different for the two sites. At the university clinic, patients aged 6–25 years were eligible, whereas at the municipal clinic, eligibility was limited to those aged 6–16 years. This difference was made to ensure inclusion of as many children and adolescents as possible knowing that the clinics had different patient populations with an older clientele at the university clinic. All participants needed dental treatment including tooth extraction, removal of supernumerary teeth, application of orthodontic appliances, caries treatment, or similar procedures. Exclusion criteria were moderate to severe intellectual disability, inability to understand Norwegian language, or planned use of benzodiazepines during treatment.

In addition, for participants below 16 years of age, one caregiver was included to answer questionnaires in addition to the child to gain insight into the parent perspective.

### Recruitment

Participants were recruited between September 2024 and February 2025, with a target of 60 individuals per site, resulting in a total sample size of 120. Half of the participants were included in the control group and the other half in the intervention group. At the university clinic, potential participants were informed about the study during pre-examination a few days before the planned dental procedure. At the municipal clinic, participants were informed about the study via a website link included in the text message appointment notification, sent at least 7 days prior to their dental visit. On the day of treatment, dedicated personnel at each study site (not dentists) provided oral and written information and obtained consents. Participants and caregivers had the opportunity to ask questions before signing consent. The given information stated that the study aimed to evaluate a communication tool designed to signal discomfort during dental treatment. It was emphasized that participation was voluntary, that the tool was intended as a supplement to usual communication methods, and that responses would be anonymized and would not affect treatment. Both intervention and control groups received identical information prior to enrolment, without disclosure of group allocation.

### Study design

**Figure 1:**
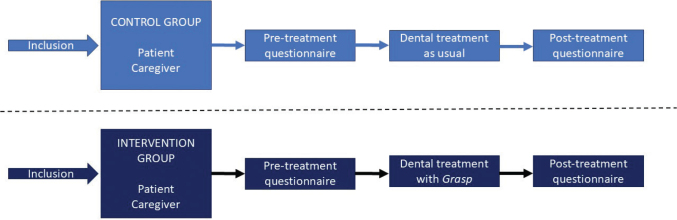
Study design. Control group recruitment was completed before the intervention group to prevent changes in standard communication due to experience with the *Grasp* intervention.

We employed a two-site controlled design where participants were randomly allocated to either the intervention group or the control group based on their assigned appointment times. At each site, the control group was enrolled before the intervention group to avoid influencing the healthcare personnel’s standard communication practices through prior experience with the *Grasp* intervention (Figure 1).

### Intervention

**Figure 2:**
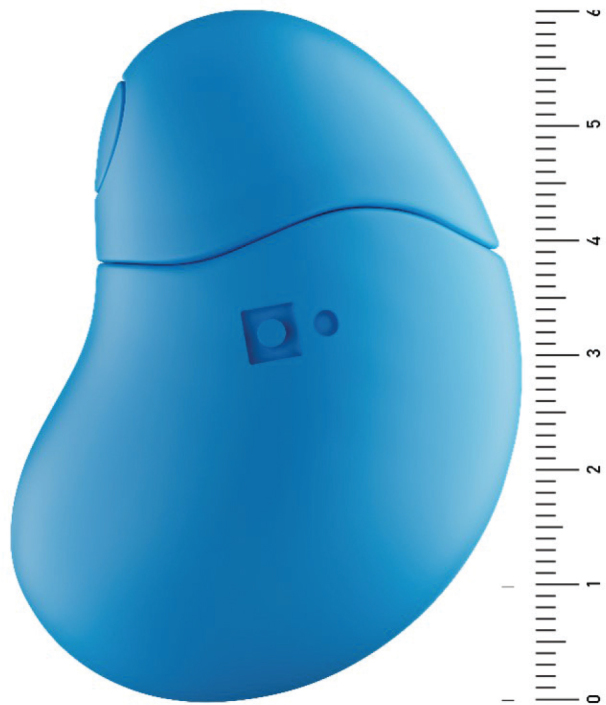
*Grasp* device with a centimeter ruler.

**Figure 3:**
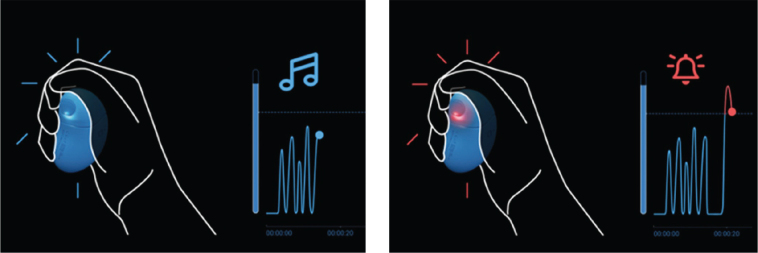
Illustration of the *Grasp Aware* feedback system. Participants were instructed to convey feelings during dental treatment by squeezing the *Grasp* device at different levels. Visual (curve fluctuations) and auditory (music or alarm) outputs corresponded to the intensity of squeezes, reflecting the participant’s discomfort level.

*Grasp* consists of hardware and software manufactured by the Grasp AS company, Norway (www.grasp.global). The concept of using Grasp in dental settings was initiated by Grasp AS and developed in collaboration with clinicians and researchers. The hardware is a handheld bean-shaped device containing a force-sensing resistor, a soft silicone inner plumb and outer layer (Figure 2). Squeezing *Grasp* reduces resistance and generates a voltage output, recorded every 0.2 seconds and reported to the software via Bluetooth. The *Grasp* device is CE-marked and approved as a medical device for symptom reporting (ID: NO918873724/0943-55311, The Norwegian Medical Products Agency). In this study all units were version UDI-DI 07073375000053. The software for the intervention was an application called *‘Grasp Aware’*, where squeezes on the *Grasp* device generated a visual output on a tablet and an auditory output from a connected speaker. The visual output was a curve illustrating the strengths of the squeeze (Figure 3), and the auditory output was music at two different levels in addition to an alarm signal. Light squeezes on the device were depicted as smaller fluctuations on the curve accompanied by a slow and calm melody. Harder squeezes on the device resulted in higher fluctuations on the curve and the sound changed to a faster and more intense melody. At the last level with the hardest squeezes, the curve turned red, and the sound changed to an alarm signal. No squeezes on the device, represented no discomfort. Weaker squeezes signaled that the participant had some discomfort, but at a manageable level. Harder squeezes represented increasing discomfort, and activating the alarm represented significant discomfort for the participant and a need of a pause in treatment. The cutoffs for the different levels were individually calibrated to user preference before the treatment started. Dentists were instructed to react on participants’ signals. Upon recognizing signs of discomfort, they were to adjust the treatment and observe if the participants’ discomfort level decreased. If the dentist heard a stop signal, the dentist should pause the treatment, check on the patient, and plan for the further treatment approach.

Before the dental treatment, participants in the intervention group were instructed in the use of the *Grasp* system in a standardized manner by study personnel. The participants practiced using the *Grasp* device to experience the outputs corresponding to various squeeze levels. It was specified that the *Grasp* intervention was a supplement to other ways of communication preferred by the participant during the dental treatment (i.e. talking, making sounds, raising a hand, etc.). The participants in the intervention group were able to use the device during the entire treatment holding it in their hands.

### Standard care

Participants in the control group received routine dental treatment according to usual clinical practice. This included verbal communication and commonly used non-verbal signals, such as raising a hand to indicate a need to pause. No additional devices or interventions were introduced for this group.

### Data

All participants were invited to answer purpose-made questionnaires before and after the treatment considering communication during the dental procedures. In addition, caregivers of participants under 16 years were invited to complete separate questionnaires to provide insights into the parent perspective. The pre-treatment questionnaire included four questions. The post-treatment questionnaire included three questions, and these were considered the outcome of this study. In the pre-treatment questionnaire, they were first asked to rate how important they found it to be able to stop the treatment. The final three pre-treatment questions were similar to the three post-treatment questions, hence, rating baseline and follow-up score on (1) how easy they expect to find/found it to speak up during dental treatment, (2) how confident they were in expecting/finding that the dentist would understand when to stop during treatment, and (3) how confident they were in expecting/finding that the dentist would understand how the participant was feeling during treatment. Answers were given on a 1–10 scale, where 1 represented not important/difficult/not confident and 10 represented important/easy/confident. In addition, data on age, sex, type of treatment, and use of local anesthesia were collected for each participant. For the caregivers, data on age and sex was collected. All questionnaires were filled out on paper and subsequently entered into data capture systems.

### Statistical analyses

Descriptive statistical analyses were performed to compare intervention and control groups. Continuous variables are reported as means with standard deviation or as medians with interquartile range (IQR). Differences were analyzed using the Wilcoxon rank-sum test for non-parametric data, with median differences estimated using the Hodges-Lehmann estimator. Categorical variables are reported as proportions and were compared using Pearson’s chi-square test or Fisher’s exact test, as appropriate based on cell frequencies. Analyses were carried out for the entire group as well as for each study site.

Assuming that the intervention and control groups did not differ at baseline, we handled the data like in a randomized design and compared groups through regression analyses with adjustment for baseline scores [17]. Hence, multiple linear regression analyses were performed to examine the impact of the intervention. Crude analyses were performed by entering covariates one by one. Thereafter, we entered baseline scores, age and sex in the same model.

No formal sample size calculation was undertaken, as the anticipated effect size of the intervention was unknown. The target of approximately 60 participants per group was based on feasibility considerations and sample sizes reported in similar studies [18, 19].

All reported confidence intervals (CI) are 95% CI. *p*-values ≤ 0.05 were regarded as significant. Statistical analyses were done in *R* version 4.2.3 [20].

### User involvement

The Youth Council at Haukeland University Hospital in Bergen, Norway, and practicing dentists were consulted during the preparatory phase of the study. Their input helped ensure that the questionnaires were age-appropriate and clinically relevant. While their feedback informed wording and usability, it did not lead to changes in the study objectives or overall design.

### Ethics

The study was approved by the Regional Committee for Medical and Health Research Ethics (REK KULMU B, application no. 743576) and conducted in accordance with the Declaration of Helsinki. All participants received oral and written information about the study, including the option to withdraw consent at any time. For participants under 16 years of age, one guardian provided consent on behalf of the child.

## Results

A total of 121 participants were included. At the university clinic, 60 participants were included (58% females) with 30 participants in both the control and the intervention group. At the municipal clinic, 61 participants were included (46% females) with 30 in the control group and 31 in the intervention group. The mean age for participants at the university clinic was 20.6 years (range 13–24), and the mean age at the municipal clinic was 12.5 years (range 6–16). The intervention and control group did not differ in age, sex, type of interventions, or use of local anesthesia (Table 1), neither for the total sample nor within study sites. For participants under 16 years of age, one caregiver per participant was included (*n* = 43). Caregivers’ median age and sex distribution did not differ between intervention and control groups (Table 1). At the university clinic, five dentists were involved in the study, and at the municipal clinic, three dentists were involved. All dentists treated participants in both the intervention and control groups, and there were no systematic differences in how participants were allocated to the dentists.

**Table 1:** Demographic and clinical characteristics of the participants.

Variable	Control group	Intervention group	*P*-value
**Participants**			
Clinic (*N* (%))^a^			0.93
University clinic	30 (50%)	30 (49%)	
Municipal clinic	30 (50%)	31 (51%)	
Age, years (median (IQR))^b^	16.0 (12.0; 21.0)	16.0 (12.0; 21.0)	0.83
Sex (*N* (%))^a^			0.78
Female	32 (53%)	31 (51%)	
Male	28 (47%)	30 (49%)	
Procedure (*N* (%))^a^			0.20
Examination	1 (2%)	4 (7%)	
Tooth extraction	7 (12%)	6 (10%)	
Surgery	23 (38%)	26 (42%)	
Restorative treatment	22 (36%)	12 (20%)	
Other	7 (12%)	13 (21%)	
Use of local anesthesia (*N* (%))^a^			0.30
Yes	54 (90%)	51 (84%)	
No	6 (10%)	19 (16%)	
Caregivers			
Number	19	24	
Age, years (median (IQR))^b^	45.0 (43.0; 51.0)	43.0 (40.0; 47.8)	0.40
Sex (*N* (%))^a^			0.26
Female	7 (37%)	13 (54%)	
Male	12 (63%)	11 (46%)	

Notes:

a Fisher’s exact test was used for categorical variables unless otherwise specified; odds ratios with 95% confidence intervals were calculated separately.

b Mann–Whitney U-test. IQR: interquartile range, *N*: Number.

All patients and caregivers completed questionnaires before and after the dental procedure. There were no missing data in the dataset. Patients’ responses to all four pre-treatment questions did not differ between the intervention and the control group (Table 2). After treatment, the patient intervention group reported significantly greater ease with speaking up during dental treatment compared to the control group (Table 2). The other two outcome measures did not differ between groups in Wilcoxon rank-sum analyses. Stratified median scores by study site are presented in Table 2.

**Table 2:** Patients’ responses to questionnaires before and after dental procedure.

Questionnaire item		Total			University Clinic			Municipal Clinic		
		Control Median [IQR]	Intervention Median [IQR]	*P*-value	Control Median [IQR]	Intervention Median [IQR]	*P*-value	Control Median [IQR]	Intervention Median [IQR]	*P*-value
Before	How important is it for you to know that you can say stop while the dentist is working inside your mouth?	8.0 [7.0; 10.0]	9.0 [7.0; 10.0]	0.77	9.0 [7.0; 10.0]	8.5 [7.2; 10.0]	0.94	8.0 [5.2; 10.0]	9.0 [6.0; 10.0]	0.67
	How easy do you find it to speak up when the dentist is working inside your mouth?	7.5 [5.0; 9.0]	6.0 [5.0; 8.0]	0.41	6.0 [4.2; 8.0]	6.0 [6.0; 8.0]	0.44	8.0 [7.0; 9.8]	7.0 [5.0; 9.0]	0.08
	How confident are you that the dentist understands when you want him or her to stop?	8.0 [6.0; 9.0]	8.0 [7.0; 9.0]	0.48	7.0 [5.0; 9.0]	8.0 [7.0; 8.8]	0.41	9.0 [7.2; 10.0]	8.0 [7.0; 9.5]	0.07
	How confident are you that the dentist will understand how you are feeling during your dental treatment today?	8.0 [5.8; 9.0]	8.0 [7.0; 9.0]	0.93	7.0 [4.2; 9.0]	8.0 [7.0; 9.0]	**0.03**	9.0 [8.0; 10.0]	8.0 [6.5; 9.0]	**0.036**
After	How easy did you find it to speak up when the dentist was working inside your mouth today?	9.0 [8.0; 10.0]	10.0 [9.0; 10.0]	**0.048**	9.0 [8.0; 10.0]	10.0 [9.0; 10.0]	**0.008**	10.0 [9.0; 10.0]	10.0 [8.5; 10.0]	0.87
	How confident were you that the dentist understood when you wanted him or her to stop during your dental treatment today?	10.0 [9.0; 10.0]	10.0 [9.0; 10.0]	0.31	10.0 [8.0; 10.0]	10.0 [9.2; 10.0]	**0.03**	10.0 [9.0; 10.0]	10.0 [9.0; 10.0]	0.44
	How confident are you that the dentist understood how you were feeling during your dental treatment today?	9.0 [8.0; 10.0]	10.0 [9.0; 10.0]	0.12	9.0 [8.2; 10.0]	10.0 [9.0; 10.0]	0.2	9.0 [8.0; 10.0]	10.0 [9.0; 10.0]	0.38

Notes: Answers were given on a 1–10 scale, where 1 represented not important/difficult/not confident and 10 represented important/easy/confident. The Wilcoxon rank-sum test was used to test difference between intervention groups. IQR: interquartile range. Bold values indicate *P*-values below 0.05.

**Figure 4:**
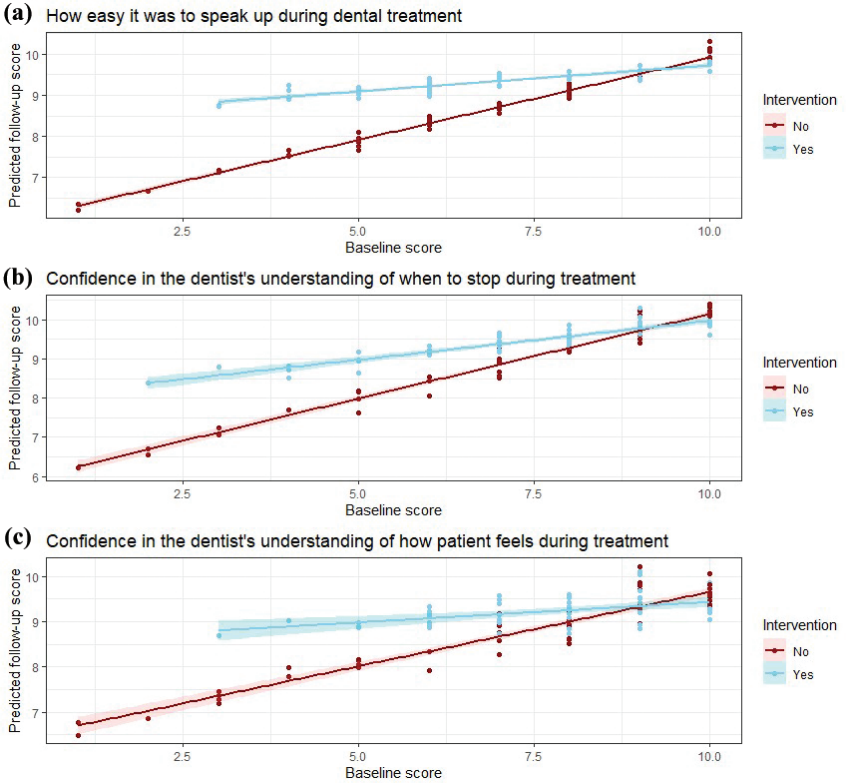
Multiple linear regression analyses for post-treatment responses to three questions regarding (a) how easy they expect to find/found it to speak up during dental treatment, (b) how confident they were in expecting/finding that the dentist would understand when to stop during treatment, and (c) how confident they were in expecting/finding that the dentist would understand how the patient was feeling during treatment. The outcomes are adjusted for age, sex, baseline score, and the interaction term between intervention and baseline score.

Multiple linear regression analyses were conducted for all three outcome measures to assess the effects of the intervention, baseline scores, and their interaction on follow-up outcomes. All models were adjusted for age and sex (Table 3). The intervention group had significantly higher follow-up scores on all three outcome measures compared to the control group (β = 2.10–2.54, *p* < 0.05). The baseline score was a significant predictor of the follow-up score for all three outcome measures, indicating that each unit increase in baseline score was associated with a corresponding increase on follow-up score. The interaction term between the intervention and baseline score was statistically significant for the questions regarding ease with speaking up and confidence that the dentist knew when the participant wanted him/her to stop. Hence, for participants with lower baseline scores, the positive impact of the intervention on follow-up scores was more pronounced (Figure 4).

**Table 3:** Unadjusted and adjusted estimates for predictors of patients’ ratings of communication-related outcomes following dental procedures.

Quetionnaire item	Unadjusted estimate	95% CI	*P*	Adjusted estimate	95% CI	*P*
**1. How easy did you find it to speak up when the dentist was working inside your mouth today?**						
Intervention	**0.61**	0.12; 1.12	**0.02**	**2.54**	1.09; 4.00	**< 0.001**
Baseline score	0.29	0.18; 0.39	**< 0.001**	**0.40**	0.27; 0.53	**< 0.001**
Interaction baseline score*intervention	-	-	-	**–0.27**	**–**0.48; **–**0,07	**0.009**
Age	**–**0.03	**–**0.09; 0.0	0.17	0.00	**–**0.04; 0.05	0.96
Sex (male)	0.02	**–**0.49; 0.5	0.95	0.04	**–**0.39; 0.47	0.85
**2. How confident are you that the dentist understood when you wanted them to stop during your dental treatment today?**						
Intervention	**0.38**	**–**0.09; 0.8	0.11	**2.10**	0.67; 3.53	**0.004**
Baseline score	0.34	0.25; 0.42	**< 0.001**	**0.43**	0.31; 0.55	**< 0.001**
Interaction baseline score*intervention	-	-	-	**–0.23**	**–**0.41; **–**0.04	**0.02**
Age	**–**0.05	**–**0.09; 0.00	0.06	**–**0.09	**–**0.17; 0.00	0.75
Sex (male)	**–**0.12	**–**0.59; 0.34	0.60	**–**0.14	**–**0.52; 0.24	0.46
**3. How confident are you that the dentist understood how you were feeling during your dental treatment today?**						
Intervention	0.43	**–**0.07; 0.93	0.09	**2.14**	0.23; 4.06	**0.03**
Baseline score	**0.26**	0.16; 0.38	**< 0.001**	**0.32**	0.19; 0.46	**< 0.001**
Interaction baseline score*intervention	-	-	-	**–**0.24	**–**0.48; **–**0.01	0.06
Age	**–**0.03	**–**0.08; 0.02	0.22	**0.00**	**–**0.05; 0.05	0.97
Sex (male)	**–**0.06	**–**0.57; 0.44	0.80	0.00	**–**0.46; 0.45	0.99

Note: Unadjusted estimates are from simple linear regression models for each predictor. Adjusted estimates are from multiple linear regression models including intervention group, baseline score, the interaction between baseline score and intervention group, age, and sex. Adjusted model *R*²: Outcome 1 = 0.27, outcome 2 = 0.35, outcome 3 = 0.17. CI: Confidence interval, IQR: interquartile range. Bold values indicate *P*-values below 0.05.

* indicates the interaction term between baseline score and intervention.

Caregivers’ questionnaires responses were analyzed for median differences (Table 4). The control group had higher median scores on three of the four pre-treatment questions, however, there were no differences between the groups’ median scores post-treatment. In multiple linear regression analyses adjusted for baseline score, age and sex of the child, and the interaction term between baseline score and intervention, the intervention had a significant effect on caregivers’ confidence that the dentist understood how their child was feeling during treatment (β 3.40, 95% CI 1.02–5.79, *p* = 0.006). In this model, the baseline score and the interaction term between baseline score and intervention were also both statistically significant (Table 5). The other outcome measures did not show a significant intervention effect in regression analyses.

**Table 4:** Caregivers’ responses to questionnaires before and after dental procedure.

Quetionnaire item		Controls Median [IQR]	Intervention Median [IQR]	*P*
Before	How important is it for you that your child can say stop during dental treatment?	10.0 [8.0; 10.0]	10.0 [8.0; 10.0]	0.84
	How easy do you think it is for your child to speak up when you are performing dental treatment?	7.0 [5.8; 9.0]	5.5 [4.0; 7.0]	**0.046**
	How confident are you that the dentist will understand when your child wants the dentist to stop?	9.0 [8.0; 10.0]	8.0 [5.5; 9.0]	**0.021**
	How confident are you that the dentist recognizes how your child is feeling when you are performing dental treatment?	8.0 [7.8; 10.0]	7.0 [6.0; 8.0]	**0.008**
After	How easy do you think it was for your child to speak up during the dental treatment today?	10.0 [8.8; 10.0]	9.0 [9.0; 10.0]	0.63
	How confident were you that the dentist understood when your child wanted the dentist to stop during the dental treatment today?	10.0 [9.0; 10.0]	10.0 [9.0; 10.0]	0.89
	How confident were you that the dentist recognized how your child was feeling during dental treatment today?	10.0 [9.0; 10.0]	10.0 [9.0; 10.0]	0.91

Notes: Answers were given on a 1–10 scale, where 1 represented not important/difficult/not confident and 10 represented important/easy/confident. The Wilcoxon rank-sum test was used to test difference between intervention groups. IQR: interquartile range. Bold values indicate *P*-values below 0.05.

**Table 5:** Unadjusted and adjusted estimates for predictors of caregivers’ ratings of communication-related outcomes following dental procedures.

Quetionnaire item	Unadjusted estimate	95% CI	*P*	Adjusted estimate	95% CI	*P*
**1. How easy do you think it was for your child to speak up during the dental treatment today?**						
Intervention	0.20	**–**0.52; 0.92	0.57	0.02	**–**2.27; 2.31	0.99
Baseline score	0.10	**–**0.06; 0.25	0.21	0.13	**–**0.11; 0.38	0.28
Interaction baseline score*intervention	-	-	-	0.04	**–**0.30; **–**0.38	0.83
Patient age	0.06	**–**0.12; 0.25	0.49	0.06	**–**0.13; 0.25	0.56
Patient sex (male)	0.32	**–**0.40; 1.04	0.38	0.48	**–**0.31; 1.26	0.23
**2. How confident were you that the dentist understood when your child wanted the dentist to stop during the dental treatment today?**						
Intervention	0.26	**–**0.42; 0.94	0.44	0.17	**–**3.60; 4.00	0.93
Baseline score	0.04	**–**0.14; 0.23	0.67	0.07	**–**0.31; 0.46	0.70
Interaction baseline score*intervention	-	-	-	0.02	**–**0.43; 0.47	0.93
Patient age	**–**0.03	**–**0.20; 0.15	0.76	**–**0.03	**–**0.22; 0.15	0.71
Patient sex (male)	**–**0.13	**–**0.55; 0.81	0.70	**–**0.02	**–**0.62; 0.93	0.70
**3. How confident were you that the dentist recognized how your child was feeling during dental treatment today?**						
Intervention	0.19	**–**0.43; 0.81	0.54	**3.40**	**1.02; 5.79**	**0.006**
Baseline score	**0.22**	**0.07; 0.37**	**0.005**	**0.48**	**0.26; 0.71**	**< 0.001**
Interaction baseline score*intervention	-	-	-	**–0.37**	**–0.68; –0.07**	**0.017**
Patient age	**–**0.09	**–**0.24; 0.07	0.27	**–**0.11	**–**0.24; 0.02	0.18
Patient sex (male)	**–**0.12	**–**0.49; 0.74	0.69	0.24	**–**0.30; 0.79	0.36

Note: Unadjusted estimates are from simple linear regression models for each predictor. Adjusted estimates are from multiple linear regression models including intervention group, baseline score, the interaction between baseline score and intervention group, patient age, and patient sex. Adjusted model *R*²: Outcome 1 = -0.01, outcome 2 = -0.09, outcome 3 = 0.29. CI: Confidence interval, IQR: interquartile range. Bold values indicate *P*-values below 0.05.

* indicates the interaction term between baseline score and intervention.

## Discussion

We explored the use of *Grasp*, a real-time, non-verbal communication tool, during dental procedures for children and youths. The results indicated that the intervention enhanced patients’ perceived ability to express discomfort and improved their confidence in being understood by the dentist. These effects were particularly evident among participants with lower baseline expectations of communication success, suggesting that *Grasp* may be especially beneficial for individuals who initially feel less empowered in the dental setting.

Previous research has demonstrated that visual aids, stop signals, and tactile interventions enhance communication and mitigate anxiety in pediatric and adult dental settings [14, 15, 21]. The integration of stop signals such as hand raising may offer patients a degree of control during treatment, thereby alleviating distress and enhancing cooperation [16]. In this regard, the findings of this study are broadly consistent with existing literature. Notably, however, we identified only a few studies investigating tools that provide elements of the dynamic, multimodal feedback offered by *Grasp* [13, 21]. The use of tangible input combined with real-time visual and auditory feedback may augment clinicians’ situational awareness and responsiveness, enabling a more continuous and nuanced exchange of information compared to conventional communication methods. This multimodal strategy is supported by evidence from cognitive science indicating that perception, decision-making, and skill acquisition are often enhanced when stimuli are presented through multiple sensory modalities [22, 23].

The significant interaction between baseline scores and intervention effects across all three outcome measures highlights the importance of tailoring communication strategies to individual needs. For participants who already felt confident in their ability to communicate, the added benefit of *Grasp* was limited. Yet, for those with lower initial confidence, the tool appeared to offer a meaningful improvement in perceived communication and control. This finding aligns with previous research emphasizing the role of patient agency and individualized care in reducing dental anxiety and improving treatment outcomes [11, 24]. One possible explanation is that having a clear and reliable way to signal discomfort – and seeing that the dentist responds – can give patients a stronger sense of control during treatment. This experience may also reinforce self-efficacy – defined as confidence in one’s ability to execute actions and influence outcomes – which is considered important for coping in stressful situations [25]. For participants who were uncertain, the tool provided reassurance and a way to act effectively during the procedure.

Unexpectedly, caregivers in the control group reported higher median scores on three of the four pre-treatment questions compared to the intervention group (Table 4). The differences may reflect random variation in a relatively small sample. Importantly, post-treatment scores did not differ between groups, and regression analyses revealed a significant positive effect of the intervention on caregivers’ confidence that the dentist understood how their child was feeling. These findings suggest that the intervention had a meaningful impact, even if not fully captured by median comparisons alone.

### Strengths and limitations

The strengths of the study are its controlled design and inclusion in two different clinical settings. However, the study has limitations. To assess effects on communication, we used purpose-made questionnaires that have not been evaluated with respect to psychometric properties. The use of 1–10 ratings was pragmatic but may be prone to ceiling effects, which may have attenuated unadjusted median differences. The reliance on self-reported measures may be subject to response bias, particularly in a clinical setting where social desirability may influence answers. Novelty bias, the tendency for an intervention to appear better when it is new, may have affected the results [26]. Furthermore, the sequential allocation of control and intervention groups, while necessary to avoid contamination, may have introduced temporal bias [26]. Another potential limitation is that dentists were aware of the study’s focus on communication and control, which may have influenced their behavior during treatment. In future studies, clinicians’ perspectives should be explored in greater depth, using designs that include a larger and more diverse sample of dentists. No long-term data were collected, limiting conclusions about the intervention’s repeated and lasting effects. Further investigations are warranted to validate our findings, with particular emphasis on assessing its efficacy among patients experiencing dental fear and anxiety.

## Conclusions

In conclusion, the use of *Grasp* as a non-verbal communication tool during dental procedures appeared to improve patients’ perceived ability to express discomfort and enhance their confidence in being understood by clinicians – particularly among those with lower baseline expectations. These findings support the integration of real-time, multimodal communication aids in dental practice. Future research should explore the long-term impact of such tools on dental fear, treatment outcomes, and patient satisfaction, as well as their applicability in other healthcare contexts.

## Authors’ contributions

Elisabeth Ørskov Rotevatn: Conceptualization; methodology; formal analysis; writing – original draft, review and editing.

Emilie Stensaker Paz: Data collection; writing – review and editing.

Louise Sandal Løkeland: Conceptualization; writing – review and editing.

Frode Guribye: Conceptualization; supervision; writing – review and editing.

Grete Olin Engan, David N Breidablik Vatne, and Unnur Bergmann: Patient screening and enrolment; data collection; writing – review and editing.

Lars Jørgen Rygh, Cecilie Gudveig Gjerde, and Torgils Lægreid: Conceptualization; methodology; supervision; writing – review and editing.

Mette Engan: Conceptualization; methodology; formal analysis; supervision; writing – original draft, review, and editing.
